# Single crystal, Hirshfeld surface and theoretical analysis of methyl 4-hydroxybenzoate, a common cosmetic, drug and food preservative—Experiment versus theory

**DOI:** 10.1371/journal.pone.0239200

**Published:** 2020-10-06

**Authors:** Abeer Sharfalddin, Bambar Davaasuren, Abdul-Hamid Emwas, Mariusz Jaremko, Łukasz Jaremko, Mostafa Hussien

**Affiliations:** 1 Department of Chemistry, Faculty of Science, King Abdulaziz University, Jeddah, Saudi Arabia; 2 Core Labs, King Abdullah University of Science and Technology (KAUST), Thuwal, Saudi Arabia; 3 Biological and Environmental Science and Engineering (BESE), King Abdullah University of Science and Technology (KAUST), Thuwal, Saudi Arabia; 4 Department of Chemistry, Faculty of Science, Port Said University, Port Said, Egypt; Beijing Foreign Studies University, CHINA

## Abstract

Methyl 4-hydroxybenzoate, commonly known as methyl paraben, is an anti-microbial agent used in cosmetics and personal-care products, and as a food preservative. In this study, the single crystal X-ray structure of methyl 4-hydroxybenzoate was determined at 120 K. The crystal structure comprises three methyl 4-hydroxybenzoate molecules condensed to a 3D framework via extensive intermolecular hydrogen bonding. Hirshfeld surface analysis was performed to determine the intermolecular interactions and the crystal packing. In addition, computational calculations of methyl 4-hydroxybenzoate were obtained using the Gaussian 09W program, and by quantum mechanical methods, Hartree Fock (HF) and Density Functional Theory (DFT) with the 6–311G(d,p) basis set. The experimental FT-IR spectrum strongly correlated with the computed vibrational spectra (R^2^ = 0.995). The energies of the frontier orbitals, HOMO and LUMO, were used to calculate the chemical quantum parameters. The lower band gap value (ΔE) indicates the molecular determinants underlying the known pharmaceutical activity of the molecule.

## 1. Introduction

The polymorphic modifications of methyl 4-hydroxybenzoate, also known as p-oxybenzoic acid methyl ester, were first reported in the 1930s [1, 2]. Lindpaintner subsequently published six different solid forms of methyl 4-hydroxybenzoate based on their melting point [3]. The crystal structure of the first stable polymorphic modification (1) of methyl 4-hydroxybenzoate was communicated first by Lin [4] at room temperature and later at low temperatures [5–7]. We have numbered these modifications to distinguish four different modifications, which doesn’t completely match Lindpaintner’s report. In all previous reports, the methyl 4-hydroxybenzoate was recrystallized from alcoholic solvents, either ethanol or methanol, at room temperature. New metastable polymorphic modifications 2 and 4 [8, 9] were obtained from temperature-controlled sublimation experiments, while polymorph 3 was crystallized from the melt [9]. All reported polymorphs of methyl 4-hydroxybenzoate crystallize in the monoclinic crystal system: the modifications 1 and 3 crystallize with Cc (9) symmetry, while polymorphs 2 and 4 crystallize in space group P21/c (14). Interestingly, among all four modifications, only 1 has three independent molecules in the asymmetric unit, Z’ = 3, and the others are Z’ = 1 polymorphs. Gelbrich et al. reported that the presence of Z’ = 3 phase is associated with the preference of local symmetry elements in the crystal structure [9]. The methyl 4-hydroxybenzoate molecules are connected through extensive O-H⋯O hydrogen bonding-forming 1D chains. The molecular geometry and the H-bonding motif (C_1^1 (8) graph set) is identical in all reported modifications. However, the main difference between the four polymorphs is the geometry of the formed 1D chains through H-bonding [8, 9]. Nath et al. carried out 2D Hirshfeld surface analysis and IR- spectroscopic studies of polymorphs 1 and 2 to highlight the differences between the two modifications in terms of hydrogen bonding and aromatic interactions [8]. The lattice energy of all four polymorphs of methyl 4-hydroxybenzoate was calculated using various theoretical approaches and compared [9].

In this study, our main aim was to combine the available crystallographic data for 4-hydroxy-benzoate, which is isomeric to the well-known salicylic acid molecule, with the results of theoretical calculations in order to redefine the molecular structure from single crystal XRD studies and to optimize the theoretical structure. Moreover, determination of the chemical properties can be achieved by predicting and experimentally describing the obtained molecular geometry of low-weight organic compounds based on available crystal state set methods. The total structure of the molecule with two different methods, Hartree Fock (HF) and Density Functional Theory (DFT) methods, was used to study the numerous vibrational modes with their wavenumbers. Subsequently, we correlated the optimized structure with the experimental data. Moreover, we used Mulliken atomic charge analysis to test the charge distribution on the molecule. Frontier molecular orbitals (FMO) analyses were calculated to obtain the quantum parameters, chemical hardness, chemical potential, and electronegativity.

## 2. Experimental

### 2.1. Chemical and methods

Methyl 4-hydroxybenzoate was purchased from Aldrich Company and used as received. The single crystals were obtained by slowly evaporating ethanol solution at room temperature. The solid-state FT-IR spectra were recorded at room temperature in the region of 4000–450 cm^-1^ using a Perkin-Elmer spectrometer.

The single crystal X-ray diffraction measurements were carried out on a Bruker D8 Venture diffractometer equipped with microfocus (IμS) Mo Kα-source, Photon 2D detector and low temperature Oxford-Cryo system. The data collection, reduction and corrections were done using the Bruker *APEX3* software suite and *SAINT* [10]. The crystal structure was solved by *SHELXT* [11] and refined with the *SHELXL* program [12]. The graphical images were produced by *Diamond* 4 [13].

### 2.2. Computational details

The Gaussian 09 program package [14] was used to obtain all theoretical calculations. Gauss View 5.0 [15] was used to prepare the output files and to visualize the molecular structure. The organic molecule was optimized in gas phase by the Hartree-Fock (HF) method using the 6-311G(d,p) basis set. Moreover, the Density Functional Theory (DFT) was used with the B3LYP functional [16] and the 6-311G(d,p) basis set. The vibrational frequencies calculation was performed for the obtained optimized molecules at the same theory level to confirm that all stationary points are minima and do not have imaginary frequencies. Moreover, frequency values computed at these levels contain known systematic errors. Therefore, the scaling factor values 0.909 and 0.967 for HF and DFT/ B3LYP, respectively, were used to correct for anharmonicity and the neglected aspect of electron correlation [17].

The frontier molecular orbitals (FMO) were computed with single point energy using the HF and DFT method with the 6-311G(d,p) basis set. Essential quantum parameters were estimated according to known equations [18–20] as follows; energy gap (ΔE = ELUMO − E_HOMO_), absolute electronegativities (χ = −E_HOMO_ +E_LUMO_/2), absolute hardness (ɳ = E_LUMO_ − E_HOMO_/2), absolute softness (σ = 1/ɳ), chemical potentials (pi = -χ), global softness (S = 1/2ɳ), global electrophilicity (ω = π^2^/2ɳ), and additional electronic charge (ΔN_max_ = −π/ɳ).

## 3. Results and discussion

### 3.1. Crystal chemistry

**Fig 1:**
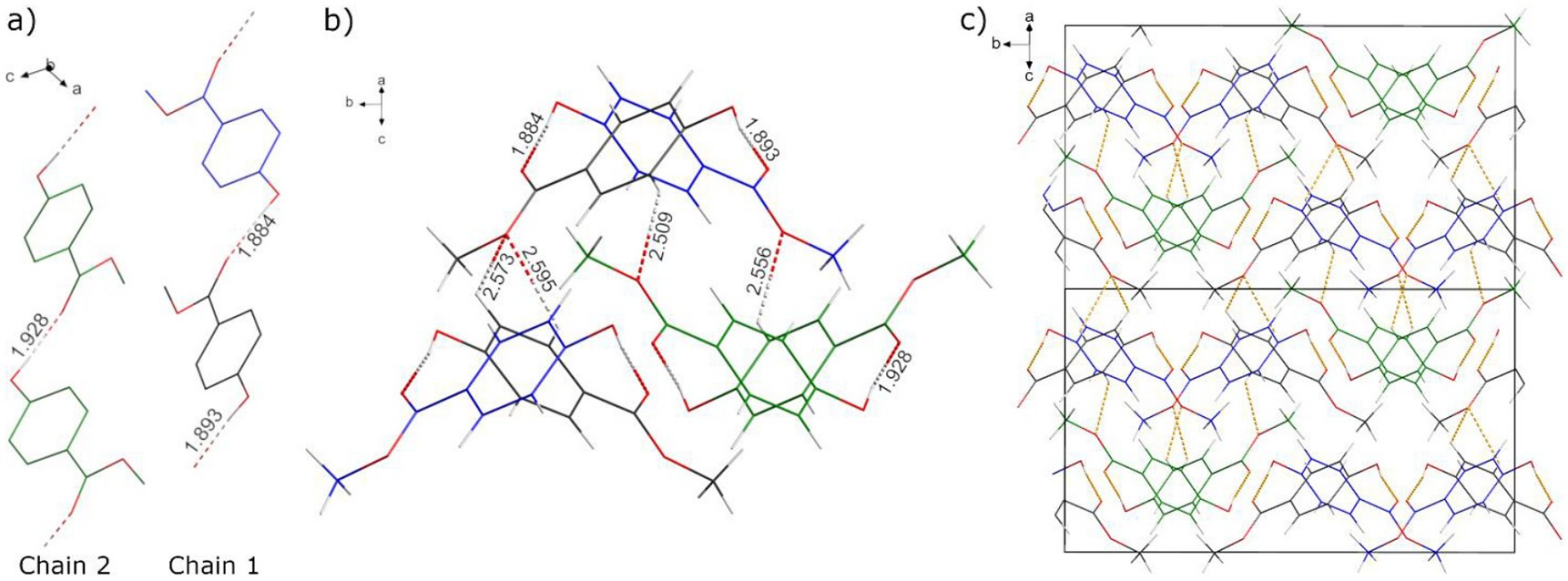
The arrangement of methyl 4-hydroxybenzoate molecules depicted by a wire-stick model. Three independent molecules are shown in black (1), blue (2), and green (3). a) 1D molecular zig-zag chains formed through O-H⋯O hydrogen bonding viewed around the *b* axis. Chain 1 is formed by alternating molecules 1 and 2, while chain 2 is composed of only molecule 3; b) 1D chains are running parallel to the [101] direction and are connected to each other via C-H⋯O H-bonding. The H-bonding distances are shown on the respective bonds; c) Supramolecular feature of the crystal structure is realized by …*AB*… stacking of the [112]∞2 layers along the [102] direction.

Block shaped, isometric transparent single crystals of the title compound were isolated from mother liquor and measured at 120 K. The methyl 4-hydroxybenzoate (methyl paraben) crystallizes with monoclinic symmetry in space group *Cc* (9) with unit cell parameters: *a* = 12.9874(7) Å, *b* = 17.2522(7) Å, *c* = 10.8435(5) Å and *β* = 119.225(2)°. All atoms were refined anisotropically and occupy general positions (4*a*). The hydrogen atoms were refined using a riding model with *U*_iso_(H) = 1.2 (aromatic) or 1.5 (methyl and hydroxyl). Table 1 summarizes the crystal data and refinement results. The crystal structure belongs to the methyl 4-hydroxybenzoate polymorph 1, mentioned in the introduction, and is identical to the crystal structure reported by Fun et al [7]. Briefly, the structure contains three crystallographically independent molecules in the asymmetric unit, Z’ = 3. The molecules are color-coded differently in Fig 1 for clarity: molecule 1—black, molecule 2—blue, and molecule 3—green. There are two different 1D chains in the crystal structure formed through O-H⋯O hydrogen bonding of methyl 4-hydroxybenzoate molecules with *D*⋯*A* distances of 2.726(2)—2.765(2) Å (Fig 1). Chain 1 is formed by alternating molecule 1 (black) and 2 (blue), while chain 2 is composed solely of molecule 3 (green).

**Table 1:** The crystallographic data and refinement details.

Chemical formula	C_8_H_8_O_3_
Molecular mass	152.14
Crystal system, space group	Monoclinic, *Cc*
Temperature (*K*)	120
*a* (Å)	12.9874 (7)
*b* (Å)	17.2522 (7)
*c* (Å)	10.8435 (5)
*β* (°)	119.225 (2)
*V* (Å^3^)	2120.34 (18)
*Z*	12
Radiation type	Mo *K*α
μ (mm^−1^)	0.11
*h*, *k*, *l*	−24 ≤ *h* ≤ 24
	−33 ≤ *k* ≤ 33
	−20 ≤ *l* ≤ 20
	0.050
*R*_int_	0.045
*R*1	0.142
*wR*2,	1.08
Goodness of fit (*S*)	15758
No. of independent reflections	301
No. of parameters Δ ρ_max_, Δρ_min_ (e Å^-3^)	0.57, −0.39

The CIF file can be obtained from the CCDC database quoting the number 2000146.

These chains are running parallel to the [101] direction and are connected by C-H⋯O hydrogen bonding, with *D*⋯*A* distances of 3.195(2)—3.337(2) Å, to form 2D layers propagating along the *b* axis. These 2D layers can be noted as [112]∞2, where the number in the bracket stands for chains 1 and 2. The supramolecular feature of the crystal structure is realized by …*AB*… stacking of [112]∞2 layers along the [102] direction. All the interatomic distances and bond angles are in the same range between the three molecules, and agree well with those in all other reported polymorphs [4, 5, 7–9].

### 3.2. Hirshfeld surface analysis

Hirshfeld surface analysis is a quantitative way to study the intermolecular interactions of the molecules in a crystal structure. Moreover, it gives details of their crystal packing behavior. Hirshfeld surfaces and fingerprint plots were mapped with Crystal Explorer 3.1 software [21]. The analysis was visualized by the normalized contact distance (d_norm_), which was obtained using a high surface resolution with a static color scale of -0.222 (red) to 1.274 Å (blue) and computed with the following Eq 1;

$$d_{norm}=\frac{d_{i}-r_{i}^{vdw}}{r_{i}^{vdw}}+\frac{d_{e}-r_{e}^{vdw}}{r_{e}^{vdw}}$$ (1)

Where d_e_ is the distance from the Hirshfeld surface to the nearest nucleus outside the surface, d_i_ is the corresponding distance to the nearest nucleus inside the surface, and *r*^*vdw*^ is the van der Waals radius of the atom [22]. The *d*_*norm*_ parameter exhibits a surface with a red-white blue color scheme [23]. Bright red spots show the intermolecular contacts less than their *vdW* radii, while the blue spots show intermolecular contacts longer than their *vdW* radii. White spots are the sum of their *vdW* radii.

**Fig 2:**
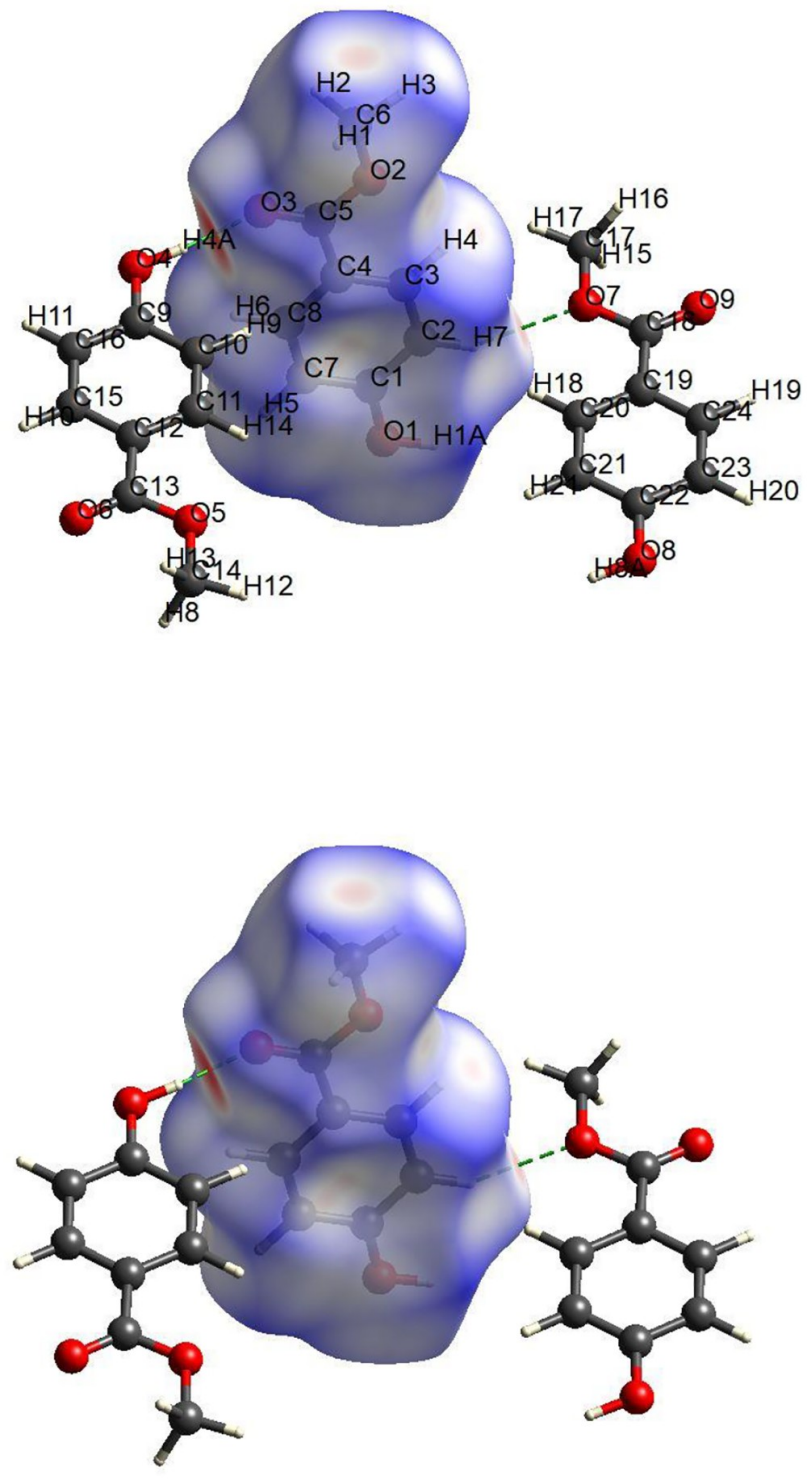
The Hirshfeld surface of the compound mapped with dnorm.

Fig 2 shows that there are two red spots, indicating hydrogen bonding contacts. The first hydrogen bond is performed by the oxygen atom (O3), which plays a role as a donor atom to the neighboring H4A atom, which acts as an acceptor atom. The second hydrogen bond is between the hydrogen atom H7 with the Oxygen O7 as the donor atom from the neighboring molecule. There are harmonious relationships between these spots and intermolecular bonding.

**Fig 3:**
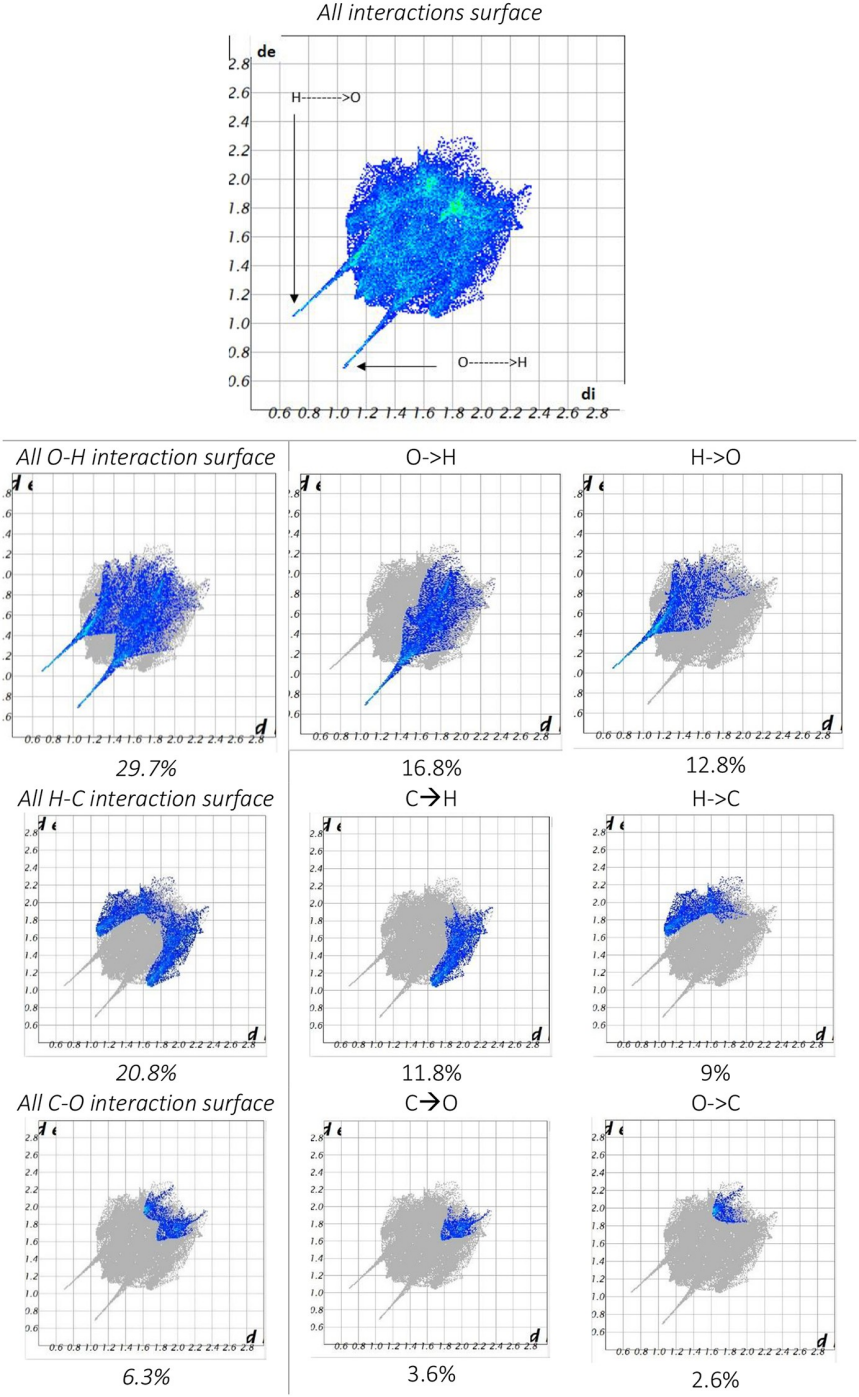
Atom-atom interactions and their contribution to the Hirshfeld surface.

The 2D representation of a Hirshfeld surface is presented in Fig 3. The O…H interactions appeared as scattered spikes in the 2D fingerprint plots with overall Hirshfeld surfaces of (16.8+12.8 = 29.7%). The contribution from the C—H contacts is represented below the pair of sharp spikes of O—H and are characterized by strong hydrogen-bonding interactions (11.8% + 9% = 20.8%) whereas the other fingerprint plots are represented in Fig 3.

### 3.3. Optimized molecular geometry

**Fig 4:**
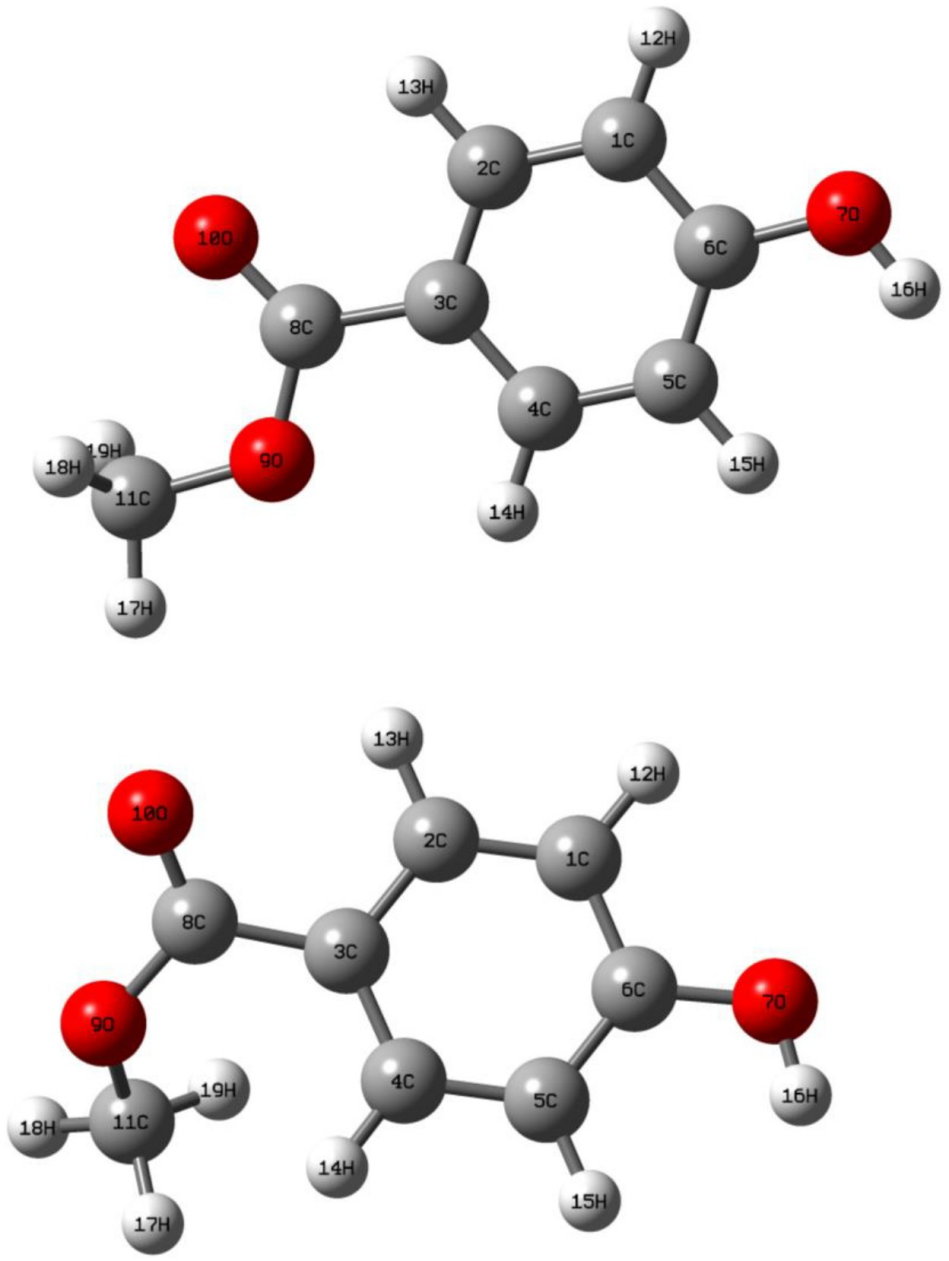
Optimized structure with atomic numbering obtained by the (a) HF and (b) DFT methods.

The optimized geometry of the methyl 4-hydroxybenzoate molecule was obtained by both HF and DFT calculations with the 6-311G (d,p) basis set, and the optimized structures are shown in Fig 4. A comparison of the optimized bond length, bond angles and dihedral angles were obtained from the HF and DFT methods with the experimental values presented in Table 2. The theoretical bond length and angles showed a slight deviation from the crystal parameters because the calculations were carried out in the gaseous phase for both HF and DFT while the experimental values were for the crystalline state. Careful analysis of the extracted data allows us to conclude that the bond length and angle calculated by the HF and DFT/6-311G(d,p) methods correlate strongly with the experimental values, with an average divergence of 0.01Å to 0.04 Å. For instance, the optimized bond of the carboxyl group O10-C8 (1.216 Å) closely matches the experimental length (1.218 Å). The experimental bond length for carboxyl O10-C8 was shorter than the ester oxygen O9-C8, and the HF (1.216/1344 Å) and DFT (1.205/1.365) methods indicated that O10-C8 is a double bond. In all experimental results, C-H bonds were shorter than those calculated by both HF and DFT. In the benzene ring, the bond length of C1-C2, C3-C4 and C5-C6 from the DFT method were in close agreement with the experimental length with minimal deviation (±0.03). This result shows that the electron delocalization occurs between those carbon atoms in the aromatic ring to make the structure more stable [24]. Moreover, the observed deviation may be due to the fact that the X- ray structure was solved in a crystalline form whereas the calculation for the optimized structures were for the isolated molecules [25].

**Table 2:** The optimized molecular geometrical parameters of methyl 4-hydroxybenzoate.

parameters	Experimental	HF	DFT
Bond length (°A)			
O7-C6	1.353	1.369	1.360
O7-H16	0.840	0.945	0.953
O10-C8	1.225	1.216	1.203
C8-O9	1.331	1.344	1.365
O9-C11	1.446	1.442	1.435
C1-C2	1.385	1.378	1.384
C1-C6	1.395	1.395	1.399
C2-C3	1.397	1.388	1.380
C1-H12	0.93	1.068	1.083
C3-C4	1.401	1.389	1.403
C2-H13	0.950	1.069	1.083
C3-C4	1.401	1.386	1.399
C3-C8	1.474	1.468	1.494
C6-C5	1.398	1.386	1.390
C4-H14	0.980	1.068	1.083
C5-H15	0.980	1.071	1.086
C11-H17	0.96	1.073	1.093
C11-H18	0.96	1.077	1.091
C11-H19	0.96	1.077	1.091
RMSD	-	0.077	0.088
Bond angle (°)			
C6-O7-H16	109.50	114.94	109.57
C8-O9-C11	116.48	119.25	122.1
O7-C6-C5	122.48	122.33	122.75
O7-C6-C1	117.47	116.88	117.38
C5-C6-C1	120.3	120.80	119.88
C2-C1-C6	120.3	119.33	119.71
C2-C1-H12	121	121.79	121.45
C6-C1-H12	119.5	118.88	118.83
C1-C2-C3	119.5	120.63	121.1
C1-C2-H13	119.5	120.44	120.31
C3-C2-H13	118.9	118.91	118.61
C2-C3-C4	122.1	119.39	118.61
C2-C3-C8	120	118.68	118.1
C4-C3-C8	120	120.93	123.1
C5-C4-C3	120.45	120.32	120.77
C5-C4-H14	120.1	120.10	119
C3-C4-H14	119.8	119.59	120.2
C4-C5-C6	119.85	119.54	119.91
C4-C5-H15	120.1	120.19	120.1
O10-C8-O9	124.58	121.64	118.8
O10-C8-C3	112.7	124.64	122.42
O9-C8-C3	109.5	113.72	118.73
O9-C11-H18	109.5	110.16	110.44
O9-C11-H18	109.5	110.17	112
RMSD	-	2.89	3.37
dihedral angles (°)			
O7—C1—C2—C3	180	179.9	179
C6—C1—C2—C3	1.2	0.018	1.2
C1—C2—C3—C4	-0.1	-0.010	-0.08
C2—C3—C4—C5	-1.2	-0.018	-1.72
C2—C3—C4—C7	177	179	177.9
C3—C4—C5—C6	1.5	0.0019	1.1
C8—C4—C5—C6	-177	-179	-177.5
C3—C4—C6—C1	-0.4	-0.0088	-0.49
O7—C6—C1—C4	-179.7	-180	-179.5
C1—C6—C5—C4	-1	-0.0123	-0.934
C11—O9—C8—O10	0.8	0.0069	0.85
C11—O9—C8—C3	-179.72	-180	-179.3
C2—C3—C8—O10	-3.4	0.0065	-3.43
C4—C3—C8—O10	175.1	179	174.71
C2—C3—C8—O9	177.1	180	177.0
RMSD	-	1.96	0.38

The related dihedral angles show that the methyl 4-hydroxybenzoate ring system is planar, as O7—C1—C2—C3, O7—C6—C1—C4 and C11—O9—C8—C3 are 180°. However, the overall dihedral angles calculated using the DFT/B3LYP method correlated well when compared with the experimental results.

In order to investigate the performance of the two methods, HF and DFT/B3LYP, for the title compound, we computed the root mean square deviation (RMSD) between the calculated bond length or angles or the dihedral angles for each method, and the experimental data. RMSD values were evaluated using the following expression [26]

$$\text{RMSD}=\sqrt{\frac{1}{\text{n}-2}\sum_{\text{i}}^{\text{n}}{\left(\text{Xexp}-\text{Xcal}.\right)}^{2}}$$ (2)

The calculated RMSD are listed in Table 2. The bond length error was very small and close to zero for both methods unless the HF method showed the best value and approximated the observed fundamental bond lengths much better than the other method. Moreover, the deviation from the experimental data of the angle values calculated using the HF method was also better than the DFT method. Interestingly, dihedral angles obtained using the DFT method were in good approximation and closer than when using the other method.

### 3.4. Mulliken atomic charge analysis

The atomic charges greatly affect molecular system properties such as the dipole moment, the electronic structure, the acidity–basicity behavior, and the vibration mode [24, 27]. Moreover, the charge distribution on the molecule is used to distinguish the donor atoms from the acceptor ligand atoms. The calculated Mulliken charge values using various levels of theory, HF and DFT with the 6-311G(d,p) atomic basis sets, are listed in Table 3 and show that one carbon (C8) has a positive charge in the geometry calculated using the HF and DFT methods because it is surrounded by two electrons withdrawing oxygen atoms, which become negatively charged atoms. Among the other negative carbon atoms, C3, C5 and C6 are more negatively charged, which indicates greater electron delocalization around the aromatic ring [24]. Despite the fact that each hydrogen atom has a positive charge, H16 has the highest positive charge (0.408 e). This is due to the binding to an atom with higher electronegativity, oxygen (O7), which could easily deprotonate in suitable medium. The high negative charge density on all oxygen atoms provide donor sites for possible coordination with metal cations. Therefore, those oxygen atoms and the positive hydrogen play crucial roles in forming the hydrogen bonding network in the crystalline state.

**Table 3:** Mulliken atomic charges for optimized geometry of methyl 4-hydroxybenzoate.

Atom label	HF/6-311++G(d,p)	DFT/6-311++G(d,p)
C1	-0.216	-0.090
C2	-0.053	-0.025
C3	-0.256	-0.241
C4	-0.028	-0.037
C5	-0.240	-0.130
C6	-0.410	0.166
O7	-0.757	-0.350
C8	0.747	0.414
O9	-0.672	-0.320
O10	-0.534	-0.302
C11	-0.170	-0.130
H12	0.197	0.113
H13	0.213	0.114
H14	0.209	0.107
H15	0.176	0.093
H16	0.408	0.251
H17	0.185	0.109
H18	0.191	0.134
H19	0.191	0.125

### 3.5. Frontier molecular orbitals (FMOs) parameters

**Fig 5:**
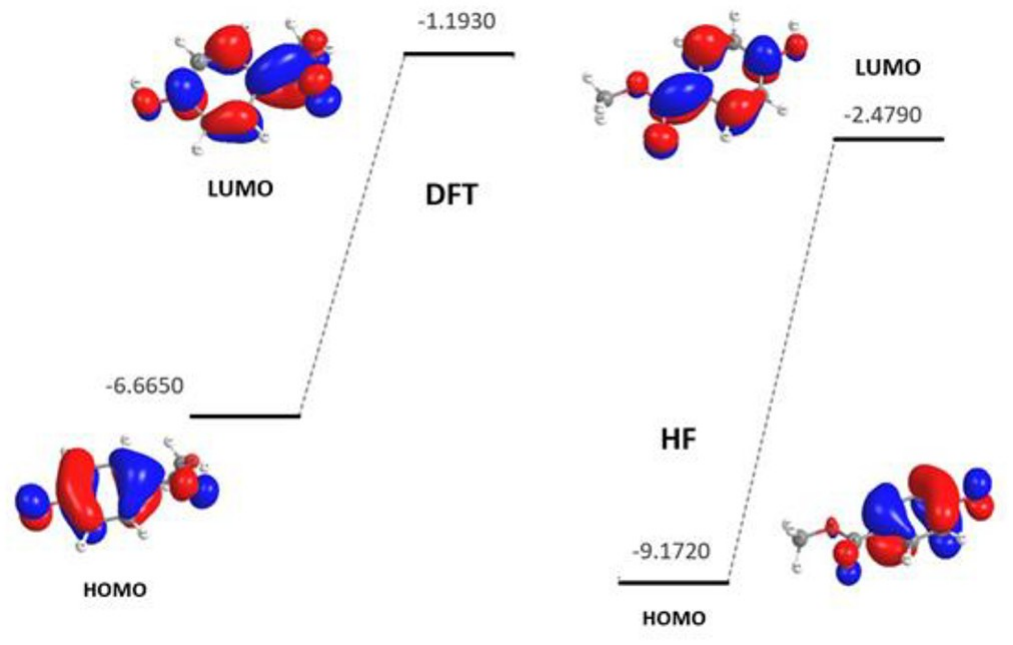
Calculated 3D of HOMOs-LUMOs plots and their energies (eV) of the compound by HF and DFT/B3LYP with 6-311G(d,p).

Frontier molecular orbitals are used to study the energies of the highest occupied molecular orbital (HOMO) and lowest unoccupied molecular orbitals (LUMO). HOMO presents the nucleophilicity of the molecule (electron donating) while LUMO illustrates the electrophilicity (electron accepting) of the molecule. The calculation of the energy of those orbitals with HF and DFT methods were at the ground state. Fig 5 shows that the charge density of the HOMO and LUMO orbitals is distributed on the phenol moiety and the ester oxygen atoms for both utilized calculation methods. The quantum chemical descriptors are summarized in Table 4. The HF/HOMO value is (-0.43 au), higher than the DFT/HOMO method (-0.24 au), where LUMO orbitals were -0.09 and -0.04 au for HF and DFT, respectively. The results revealed the abilities of the methyl 4-hydroxybenzoate molecule to donate electrons to electrophilic molecules. The energy gap (ΔE) is the difference between the HOMO and LUMO, which is used to determine computational parameters namely: chemical hardness, chemical softness, electronegativity, and the chemical potential of a molecule. The small energy gap (ΔE) indicated a high reactivity of methyl 4-hydroxybenzoate with low kinetic stability [24]. Moreover, the molecule has high softness values (S) indicating a high reactivity and an interpretation of the biological activity of the molecule [28, 29]. The negative value of the chemical potential (pi) of methyl 4-hydroxybenzoate indicates spontaneous reaction processes [30].

**Table 4:** Quantum chemical parameters for methyl 4-hydroxybenzoate calculated at the HF and DFT levels.

method	HUMO	LUMO	Δ E	x	ɳ	σ	Pi	σ	S	ω	ΔN max
DFT/ B3LYP	-6.67	-1.19	5.47	3.93	2.74	0.37	-3.93	0.37	1.37	2.82	1.44
HF	-9.17	-2.48	6.69	5.83	3.35	0.30	-5.83	1.67	1.67	2.91	1.74

### 3.6. Assignments of vibrational spectra

**Fig 6:**
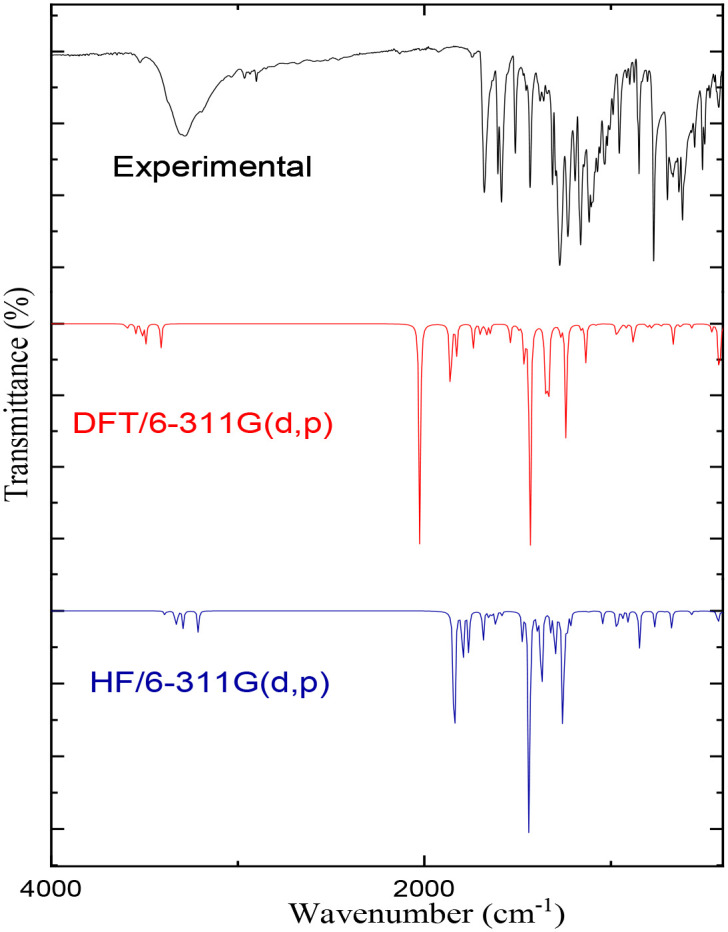
Comparative representations of FT-IR spectra for methyl 4-hydroxybenzoate.

**Fig 7:**
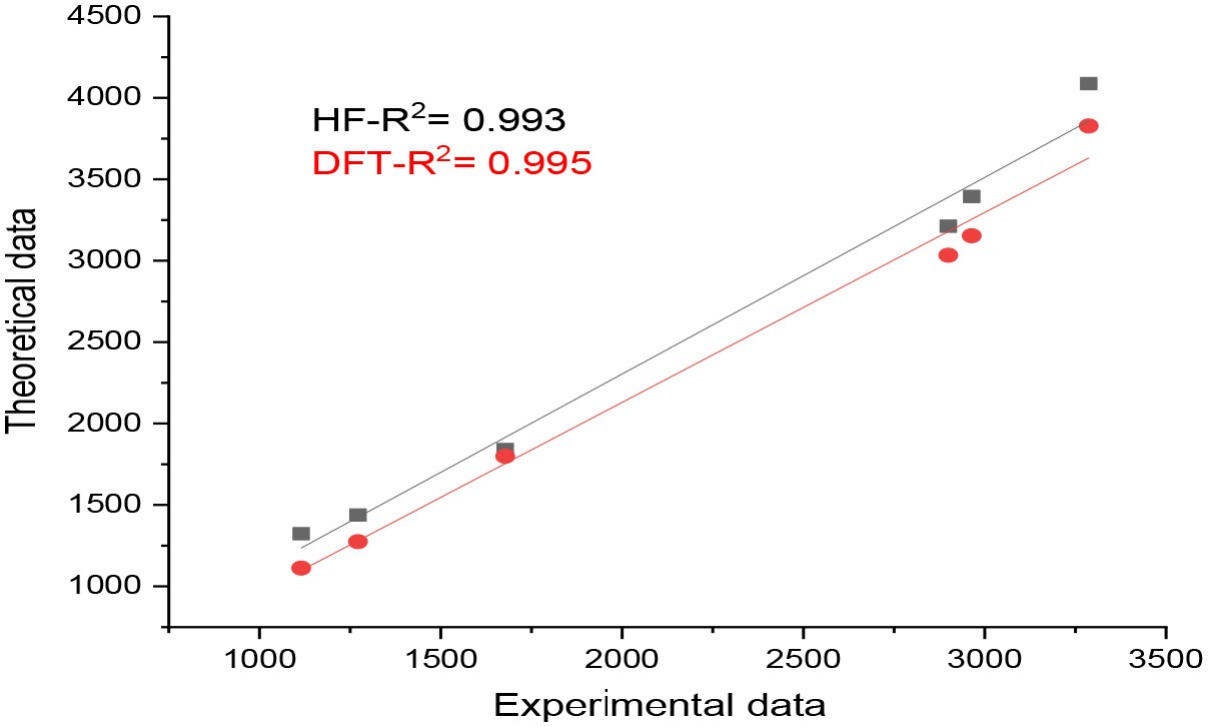
Correlation between observed and calculated IR vibrational frequencies of methyl 4-hydroxybenzoate.

Studying the vibrational spectroscopic spectrum gives a valuable description of the bonding force and the intermolecular bond strength. The FT-IR analysis for the compound was performed with solid sample in the range 400–4000 cm^-1^ and is illustrated in Fig 6 with the corresponding theoretical spectra. The essential assignment vibration bands of methyl 4-hydroxybenzoate are listed in Table 5 and are compared with the computed results of both HF and DFT/B3LYP with the 6-311G (d,p) basis sets. The OH stretching frequency appeared at 3695 and 3700 cm^-1^ for HF and DFT, respectively. The corresponding stretching band in the IR spectrum is at 3287 cm^−1^ with a broadened envelope band due to hydrogen bonding of the phenolic OH group. The hydrogen bond caused the division of the theoretical results and shifted the experimental OH peak to low frequencies [24]. The stretching vibration value of CH phenyl banding was 3040 cm^-1^ while the vibration of the methyl group was seen at 2900 cm-1. The DFT calculation results for these bands and the HF value are close to the experiments frequencies range. The most characterized band for the ester group (C = O) stretching absorbance was found at 1678 cm^-1^ in the IR spectrum. Moreover, the other observed band for C = O at 1588 cm^−1^ is due to the hydrogen bonded to ester group [31]. The HF and DFT calculation showed the C = O frequency at 1672.5 and 1731 cm^-1^, respectively. the HF value was consistent with the experimental results. The slight differences between these values and the experimental ones are mainly due to the fact that theoretical results were calculated in the gas phase. The vibrations of the other effective bands for esters group C-O-CH_3_ at 1272 cm^-1^ and CH_3_-O at 1115 cm^-1^ were in the same bond as identified by the HF method. The peaks below 1000 cm^-1^ were for out-of-plane CH bends and observed in the same range. The correlation between the experimental and theoretical results were R^2^ = 0.993 and 0.995 for HF and DFT methods, indicating a strong, reliable correlation for experimental and calculated vibrational absorbance values with the HF and DFT methods (See Fig 7).

**Table 5:** Observed and calculated IR wavenumbers for methyl 4-hydroxybenzoate.

Assignments	Experimental	HF/Scal.	DFT/Scal.
υ OH	3287	3694.9	3701.1
υ CH phenyl	3040	3068.6	3048.9
υ CH methyl	2900	2903.3	2932.7
υ C = O	1678–1588	1672.5	1731.1
υ C-O-CH_3_	1292	1292.7	1231.9
υ CH_3_-O	1115	1117.4	1075.3

## Conclusion

In an attempt to explore the optimized geometry of methyl 4-hydroxybenzoate and harmonic vibration mode, we carried out theoretical studies at the HF and DFT levels with the 6-311G (d,p) basis set, using the structural data obtained from single crystal XRD. The computed RMSD values illustrated that the bond length and the dihedral angle obtained from the single crystal structural analysis were most likely correlated to the theoretical calculation HF and DFT, respectively. Mulliken atomic charge analysis indicated that the three oxygens are donor atoms in the molecule and active sites for chemical bonding. The low value of the energy gap between HOMO and LUMO informs on the reactivities of the organic molecule and agrees with the high softness results. The collected results will be useful in further studies focusing on the coordination chemistry of methyl 4-hydroxybenzoate with metal ions and subsequent biological research.

## Supporting information

S1 File All output file for the calculation have attached in the supplementary data to this article. (RAR)

S2 File (PDF)
