# Supplementary material for: Single crystal, Hirshfeld surface and theoretical analysis of methyl 4-hydroxybenzoate, a common cosmetic, drug and food preservative—Experiment versus theory
Source: PLoS One. 2020 Oct 6;15(10):e0239200. doi: 10.1371/journal.pone.0239200 (PMC7537869; doi:10.1371/journal.pone.0239200)

checkCIF/PLATON report

Structure factors have been supplied for datablock(s) abeer1_mono_a

THIS REPORT IS FOR GUIDANCE ONLY. IF USED AS PART OF A REVIEW PROCEDURE FOR PUBLICATION, IT SHOULD NOT REPLACE THE EXPERTISE OF AN EXPERIENCED CRYSTALLOGRAPHIC REFEREE.

No syntax errors found. CIF dictionary Interpreting this report

Datablock: abeer1_mono_a

Bond precision: C-C = 0.0020 A Wavelength=0.71073

Cell:	a=12.9874(7)	b=17.2522(7)	c=10.8435(5)
	alpha=90	beta=119.225(2)	gamma=90
Temperature:	120 K		

	Calculated	Reported
Volume	2120.34(18)	2120.34(18)
Space group	C c	C c
Hall group	C -2yc	C -2yc
Moiety formula	C8 H8 O3	C8 H8 O3
Sum formula	C8 H8 O3	C8 H8 O3
Mr	152.14	152.14
Dx,g cm-3	1.430	1.430
Z	12	12
Mu (mm-1)	0.110	0.110
F000	960.0	960.0
F000’	960.60	
h,k,lmax	24,33,20	24,33,20
Nref	15804[7910]	15758
Tmin,Tmax	0.984,0.984	
Tmin’	0.984	

Correction method= Not given

Data completeness= 1.99/1.00 Theta(max)= 43.129

R(reflections)= 0.0450(11585) wR2(reflections)= 0.1424(15758)

S = 1.077 Npar= 301

The following ALERTS were generated. Each ALERT has the format
test-name_ALERT_alert-type_alert-level.
Click on the hyperlinks for more details of the test.

Alert level C

```
PLAT052_ALERT_1_C Info on Absorption Correction Method   Not Given     Please Do !
PLAT905_ALERT_3_C Negative K value in the Analysis of Variance ...     -0.605 Report
PLAT911_ALERT_3_C Missing FCF Refl Between Thmin & STh/L=    0.600         13 Report
PLAT913_ALERT_3_C Missing # of Very Strong Reflections in FCF ....         10 Note
PLAT934_ALERT_3_C Number of (Iobs-Icalc)/Sigma(W) > 10 Outliers ..          1 Check
```

Alert level G

```
PLAT007_ALERT_5_G Number of Unrefined Donor-H Atoms ..............          3 Report
PLAT019_ALERT_1_G _diffrn_measured_fraction_theta_full/*_max < 1.0      0.995 Report
PLAT128_ALERT_4_G Alternate Setting for Input Space Group  Cc              Ia Note
PLAT883_ALERT_1_G No Info/Value for _atom_sites_solution_primary .     Please Do !
PLAT912_ALERT_4_G Missing # of FCF Reflections Above STh/L=  0.600          2 Note
PLAT978_ALERT_2_G Number C-C Bonds with Positive Residual Density.         21 Info
```

```
   0 ALERT level A = Most likely a serious problem - resolve or explain
   0 ALERT level B = A potentially serious problem, consider carefully
   5 ALERT level C = Check. Ensure it is not caused by an omission or oversight
   6 ALERT level G = General information/check it is not something unexpected

   3 ALERT type 1 CIF construction/syntax error, inconsistent or missing data
   1 ALERT type 2 Indicator that the structure model may be wrong or deficient
   4 ALERT type 3 Indicator that the structure quality may be low
   2 ALERT type 4 Improvement, methodology, query or suggestion
   1 ALERT type 5 Informative message, check
```

It is advisable to attempt to resolve as many as possible of the alerts in all categories. Often the minor alerts point to easily fixed oversights, errors and omissions in your CIF or refinement strategy, so attention to these fine details can be worthwhile. In order to resolve some of the more serious problems it may be necessary to carry out additional measurements or structure refinements. However, the purpose of your study may justify the reported deviations and the more serious of these should normally be commented upon in the discussion or experimental section of a paper or in the "special_details" fields of the CIF. checkCIF was carefully designed to identify outliers and unusual parameters, but every test has its limitations and alerts that are not important in a particular case may appear. Conversely, the absence of alerts does not guarantee there are no aspects of the results needing attention. It is up to the individual to critically assess their own results and, if necessary, seek expert advice.

Publication of your CIF in IUCr journals

A basic structural check has been run on your CIF. These basic checks will be run on all CIFs submitted for publication in IUCr journals (*Acta Crystallographica*, *Journal of Applied Crystallography*, *Journal of Synchrotron Radiation*); however, if you intend to submit to *Acta Crystallographica Section C* or *E* or *IUCrData*, you should make sure that full publication checks are run on the final version of your CIF prior to submission.

Publication of your CIF in other journals

Please refer to the *Notes for Authors* of the relevant journal for any special instructions relating to CIF submission.

PLATON version of 16/04/2020; check.def file version of 09/03/2020

Datablock abeer1_mono_a - ellipsoid plot

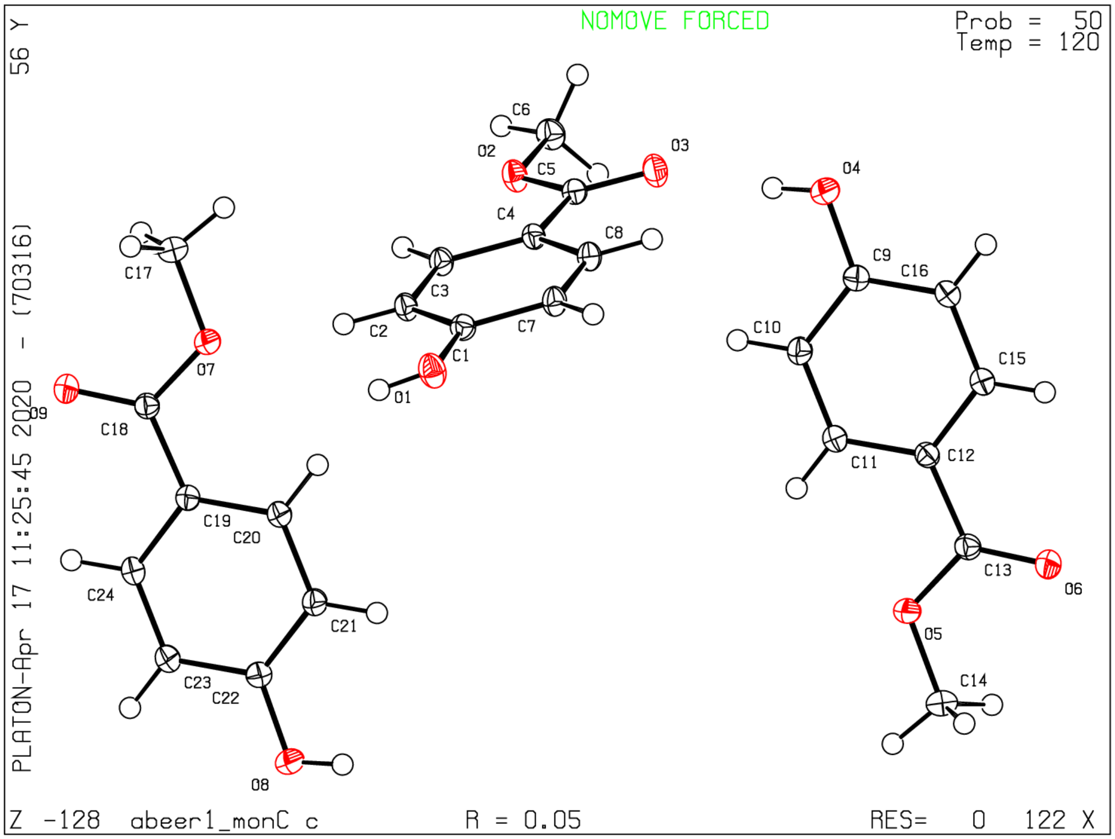

Supplement: S2 File — (PDF) [file pone.0239200.s002.pdf]
